# Predictive value of phosphorylated mammalian target of rapamycin for disease-free survival in breast cancer patients receiving neoadjuvant chemotherapy

**DOI:** 10.3892/ol.2014.2551

**Published:** 2014-09-18

**Authors:** SHUO WANG, YIQUN SUN, ANNING HE, CAIWEI ZHENG, XINYU ZHENG

**Affiliations:** 1 Department of Breast Surgery, First Affiliated Hospital, China Medical University, Shenyang, Liaoning 110001, P.R. China; 2 Department of Burns, General Hospital of Benxi Iron and Steel Company, Benxi, Liaoning 117000, P.R. China; 3 Lab 1, Cancer Institute, China Medical University, Shenyang, Liaoning 110001, P.R. China; 4 Department of Biology, Brandeis University, Waltham, MA 02453, USA

**Keywords:** phosphorylated-mammalian target of rapamycin, phosphorylated-eukaryotic translation initiation factor 4E-binding protein, neoadjuvant chemotherapy, predictive value

## Abstract

The mammalian target of rapamycin (mTOR)/eukaryotic translation initiation factor 4E-binding protein 1 (4E-BP1) pathway plays a critical role in cell growth, survival and angiogenesis, and has been demonstrated to correlate with human epidermal growth factor receptor 2 (HER2) status. Neoadjuvant chemotherapy (NAC), also known as preoperative therapy, is now well established in the treatment of inoperable locally advanced and inflammatory breast cancer. *In vitro* study has shown that mTOR inhibitors, together with cytotoxic agents, exhibit tumor cell killing activity. A number of non-randomized studies in HER2-positive trastuzumab-resistant metastatic breast cancer have revealed the antitumor activity of mTOR inhibitors when used together with standard chemotherapy plus trastuzumab. In the present study, the expression levels of phosphorylated (p)-mTOR and p-4E-BP1 were analyzed in breast cancer patients prior to and following NAC, to determine whether p-mTOR and p-4E-BP1 affect the response to NAC and the subsequent survival. Formalin-fixed, paraffin-embedded tissues representing matched pairs of core biopsy (pre-NAC) and surgical specimen (post-NAC) from 83 patients with invasive ductal carcinomas were collected. Immunohistochemistry was performed to evaluate the expression of p-mTOR and p-4E-BP1 using a semi-quantitative scoring system by two pathologists. It was found that the expression of p-mTOR and p-4E-BP1 was downregulated following NAC. The decrease in mTOR expression following NAC was found to positively correlate with HER2 expression and the reduction of tumor sizes. The high expression of p-mTOR and p-4E-BP1 in pre-NAC specimens was associated with poor disease-free survival (DFS). Furthermore, the high expression of p-mTOR in post-NAC specimens was associated with poor DFS, regardless of whether the expression was high or low in the pre-NAC specimens. In conclusion, NAC was found to decrease the expression levels of p-mTOR and p-4E-BP1. The p-mTOR expression post-NAC may potentially serve as a predictor for DFS. However, further study is required to clarify the mechanism and to evaluate the predictive value of the phosphatidylinositol 3-kinase/Akt/mTOR/4E-BP1 pathway in NAC.

## Introduction

The mammalian target of rapamycin (mTOR), a highly conserved serine/threonine protein kinase, is critical for cell growth, survival and angiogenesis (1). Members of the epidermal growth factor receptor family, including human epidermal growth factor receptor 2 (HER2), use the phosphatidylinositol 3-kinase (PI3K)/Akt/mTOR pathway to promote cell growth and survival (2). mTOR is predominantly controlled by the PI3K/Akt pathway and can be activated by Akt-mediated phosphorylation (3,4). mTOR has two main downstream messengers, the 40S ribosomal S6 kinase (S6K1) and the eukaryotic translation initiation factor 4E-binding protein 1 (4E-BP1) (5), both of which are activated via phosphorylation by phosphorylated (p)-mTOR.

The eukaryotic initiation factor 4E (eIF4E) complex is an initiation factor on the 5′ cap structure of mRNA that recruits the small ribosomal subunit to mRNA. It contains three initiation factors, eIF4E, eIF4G and eIF4A (2,6). To assemble the eIF4E complex, eIF4E first binds to the 5′ cap to recruit eIF4G and eIF4A. However, 4E-BP1 inhibits eIF4G binding to eIF4E. The p-4E-BP1 loses its ability to bind to eIF4E and allows the eIF4E complex to bind to the cap structure of mRNA (7–9), subsequently initiating the protein translation. It has been shown that eIF4E expression is associated with patient survival following anthracycline chemotherapy treatment, and the influence of eIF4E on cancer survival is modulated substantially by 4E-BP1 (10,11). The PI3K/Akt/mTOR/4E-BP1 pathway has also been found to correlate with HER2, and it may be used as a predictive marker for patient prognosis (2). Another downstream factor, S6K1, is associated with the translational machinery and has been demonstrated to predict poor prognosis in hormonal receptor-positive breast cancer patients (12,13).

Neoadjuvant chemotherapy (NAC), also known as preoperative therapy, uses chemotherapy as the initial treatment of malignant tumors, followed by surgery or other therapies. NAC is now well established in the treatment of inoperable locally advanced and inflammatory breast cancer. It is also used in operable breast cancer treatment in order to obtain clinical and pathological response to NAC, or to downstage tumors to allow breast-conserving surgery (14,15). A large number of studies have demonstrated the efficacy of NAC in primary operable and locally advanced breast cancer patients, as well as patients who have achieved pathological complete response (pCR), which is regarded as a good surrogate predictor of disease-free survival (DFS) and overall survival (OS) (16–19). However, few molecular markers are available to predict the NAC responses or survival gains. It has been demonstrated that PI3K/Akt/mTOR is commonly deregulated in human cancers (20,21), due to the mutation of PIK3CA, Akt and phosphatase and tensin homolog (PTEN), or the loss of PTEN (22–25). It has also been demonstrated that the combination of mTOR inhibitors with cytotoxic agents can exert synergistic antiproliferative activity in *in vitro* studies, irrespective of HER2 status (26). A number of non-randomized studies in HER2-positive trastuzumab-resistant metastatic breast cancer have shown the antitumor activity of mTOR inhibitors when used together with standard chemotherapy plus trastuzumab (27,28). However, a study evaluating the addition of mTOR inhibitors to paclitaxel treatment in HER2-negative patients suggested that supplementing paclitaxel treatment with everolimus did not significantly improve pCR rates compared with those of paclitaxel alone (29). A number of other clinical trials have been initiated to identify the most beneficial therapeutic strategies to include mTOR inhibitors for different patient subgroups (30).

## Materials and methods

### Patients and tissues

In total, 83 primary breast cancer patients treated with NAC at the First Hospital of China Medical University (Shenyang, China) between 2007 and 2010 were selected. Preoperative chemotherapy was performed as follows: 37 patients were administered docetaxel (75 mg/m^2^) with platinum (TP; 100 mg/m^2^) or cyclophosphamide (TC; 1.0 g) every three weeks for three to five cycles, while the other 46 patients received 5-fluorouracil (1.0 g), epirubicin (80 mg/m^2^) and cyclophosphamide (CEF; 1.0 g) every three weeks for three to four cycles. Patients were followed-up for a median of 45 months after their initial cancer surgery. Relevant clinical and pathological parameters are described in Table I. Archival formalin-fixed, paraffin-embedded breast tissues were collected from core biopsies (pre-NAC) and matched resection tissues (post-NAC). Six patients achieved pCR. All of the carcinomas had been histologically confirmed as invasive breast cancer according to the criteria of the World Health Organization (31) and the molecular subtypes of breast carcinoma were identified.

### Immunohistochemical staining

Immunohistochemical examination was performed on 4-μm formalin-fixed, paraffin-embedded sections. Briefly, following deparaffinization and rehydration, the endogenous peroxidase activity was blocked using 3% H_2_O_2_ (reagent A; UltraSensitive™ SP IHC kit; Maxim Biotech Inc., Fuzhou, China). Next, antigen retrieval was performed and normal serum was applied to the sections to block non-specific antibody binding (reagent B; UltraSensitive™ SP IHC kit; Maxim Biotech Inc.). Sections were then incubated overnight at 4°C with the primary antibodies. A goat anti-rabbit polyclonal antibody against mTOR (phospho S2448) was used for p-mTOR at a dilution of 1:500 (ab131538; Abcam, Cambridge, UK), and a goat anti-rabbit polyclonal antibody against eIF4EBP1 (phospho Thr36) was used for p-4EBP1 at a dilution of 1:200 (ab47365; Abcam). Following overnight incubation at 4°C, sections were incubated for 15 min with the secondary antibody solution (reagent C; UltraSensitive™ SP IHC kit; Maxim Biotech Inc.). The sections were then incubated with streptavidin-perosidase (reagent D; UltraSensitive™ SP IHC kit; Maxim Biotech Inc.) for 15 min and stained with 3,3-diaminobenzidine. Finally, sections were counterstained with hematoxylin for 5 min and mounted. Negative controls were processed with normal rabbit serum (Dako, Carpinteria, CA, USA) in place of the primary antibody. Positive controls were performed using breast cancer tissue sections that had shown strong staining for the respective protein during antibody optimization. This study was approved by the ethics committee of the First Affiliated Hospital (Shenyang, China) and written informed consemt was obtained from all patients.

### Evaluation of immunohistochemistry

The immunohistochemical staining results were evaluated and scored independently in a blinded manner by two pathologists. Cases of disagreement were reviewed jointly to obtain a consensus score. The score was the average of 10 distinct high-power fields observed under the 40× objective. The staining was considered positive when cytoplasmic and/or membranous staining was observed in the malignant cells, and the staining was evaluated using a semi-quantitative scoring system considering the extent and intensity. The percentage of cells stained were scored as follows: 0, no cells stained; 1, 1–10% of cells stained; 2, 11–50% of cells stained; 3, 51–80% of cells stained; and 4, >80% of cells stained. Staining intensity was scored as follows: 0, negative; 1, weak; 2, moderate; and 3, strong. The two parameters were multiplied, resulting in an individual immunoreactivity score ranging between 0 and 12 for every case. The six patients who achieved pCR were regarded as negative post-NAC.

### Statistical analysis

Statistical analyses were performed using SPSS v.19.0 (SPSS, Inc., Chicago, IL, USA). Wilcoxon signed-rank test was used to evaluate the independence between two groups of matched samples, and Mann-Whitney U test was used to assess the independence between two independent samples without any distribution assumption. Spearman’s correlation coefficients were used to reveal a correlation between two continuous variables. Receiver Operating Characteristic (ROC) curve analyses were used to select cut-off values (giving the highest combined sensitivity and specificity) to dichotomize pre- and post-NAC expression scores for the endpoint of DFS. DFS was recorded from the date of surgery to the date of relapse or last follow-up, and estimated using the Kaplan-Meier analyses. The statistical significance of the differential survival was assessed using the log-rank (score) test. Additionally, multivariate Cox regression analysis was performed taking into account the pre-NAC expression of p-mTOR and p-4E-BP1, and post-NAC expression of p-mTOR, as well as other clinicopathological features, including tumor grade, receptor status, pre-NAC tumor stage and axillary metastasis. All P-values presented are two-sided, and P≤0.05 was considered to indicate a statistically significant difference.

## Results

### p-mTOR and p-4E-BP1 are downregulated following NAC and correlate with each other in pre-NAC samples

**Figure 1:**
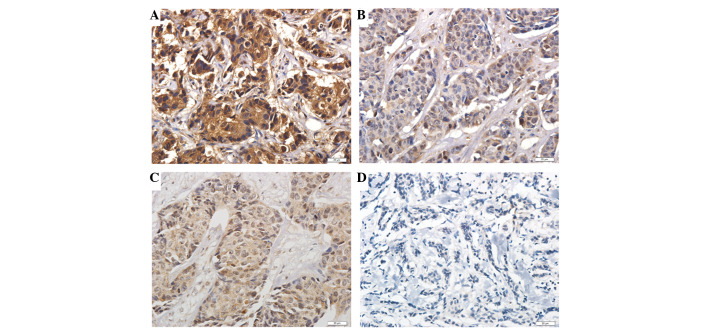
Representative staining images of phosphorylated-mammalian target of rapamycin in matched breast tumor tissues (A) pre- and (B) post-NAC chemotherapy, and phosphorylated-eukaryotic translation initiation factor 4E-binding protein (C) pre- and (D) post-NAC chemotherapy. NAC, neoadjuvant (staining intensity, A, strong; B, moderate; C, moderate; D, negative). Bar, 20 μm; magnification, ×400.

**Figure 2:**
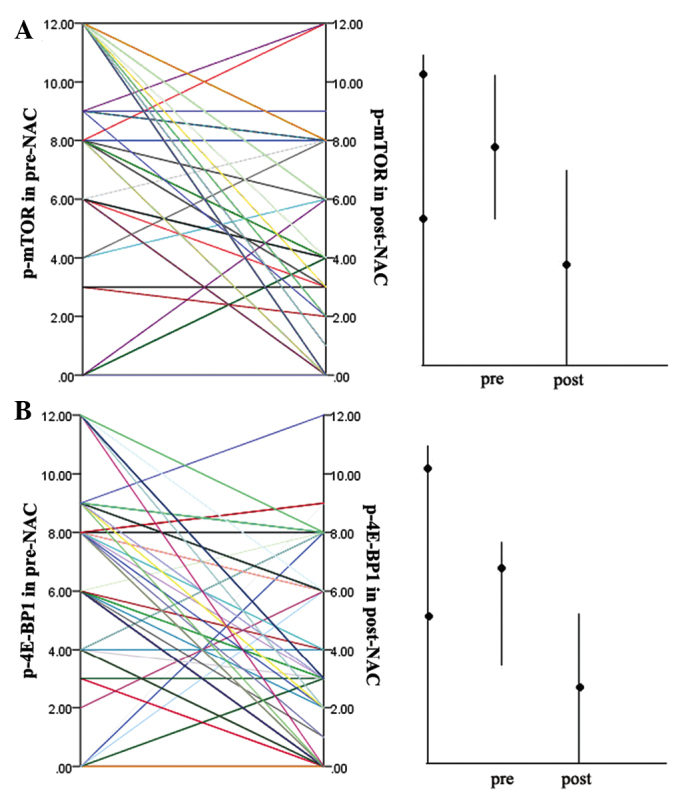
Expression levels of (A) p-mTOR and (B) p-4E-BP1 in matched breast tumor tissues pre- and post-NAC. Left panels show the distribution of the scores of evaluation of the immunohistochemistry pre- and post-NAC. Right panels show the median values (central marker) with interquartile ranges (bars) pre- and post-NAC. The median expression of p-mTOR and p-4E-BP1 were decreased signifcantly following chemotherapy. NAC, neoadjuvant chemotherapy; p-mTOR, phosphorylated-mammalian target of rapamycin; p-4E-BP1, phosphorylated-eukaryotic translation initiation factor 4E-binding protein 1.

The expression of p-mTOR and p-4E-BP1 were examined in the tumors of 83 cases of breast cancer patients. The patients who had achieved pCR were not examined post-NAC. Tumors were obtained from diagnostic biopsies pre-NAC and surgical resections post-NAC. Table I shows the clinical and pathological features of the patient cohort. The staining of p-mTOR was predominantly cytoplasmic, and present in 71/83 cases (85.5%) and 58/83 cases (69.9%) pre-NAC (Fig. 1A) and post-NAC (Fig. 1B), respectively. The p-4E-BP1 was detected in the nucleus and cytoplasm, with positive rates of p-4E-BP1 in 69/83 cases (83.1%) and 58/83 cases (69.9%) pre-NAC (Fig. 1C) and post-NAC (Fig. 1D), respectively. The scores and their distributions are shown in Fig. 2. Scores were lower for p-mTOR (51/83 cases; 61.4%) and p-4E-BP1 (56/83 cases; 67.5%) in post-NAC samples than in the matched pre-NAC samples, indicating a decrease in their expression in response to NAC. Wilcoxon signed-rank test showed significant differences between pre- and post-NAC scores (P<0.001 for p-mTOR and p-4E-BP1). It was also examined whether a correlation exists between the expression of p-mTOR and p-4E-BP1 pre- and post-NAC, as well as for the expression change following treatment (pre-NAC minus post-NAC level). The expression of p-mTOR and p-4E-BP1 were found to significantly correlate with each other in pre-NAC samples (Spearman’s ρ analyses, P=0.004), which is consistent with previous studies (2,32,33). However, the two factors were not found to correlate with each other in post-NAC samples, indicating that chemotherapy may have changed the expression of the two factors to some degree. No significant correlations were found between the changes of p-mTOR and p-4E-BP1 following NAC. As would be expected, the pre- and post-NAC levels were significantly associated with the expression change of the respective factor.

### Decrease of p-mTOR expression following NAC positively correlates with HER2 expression and diminishing tumor size

**Figure 3:**
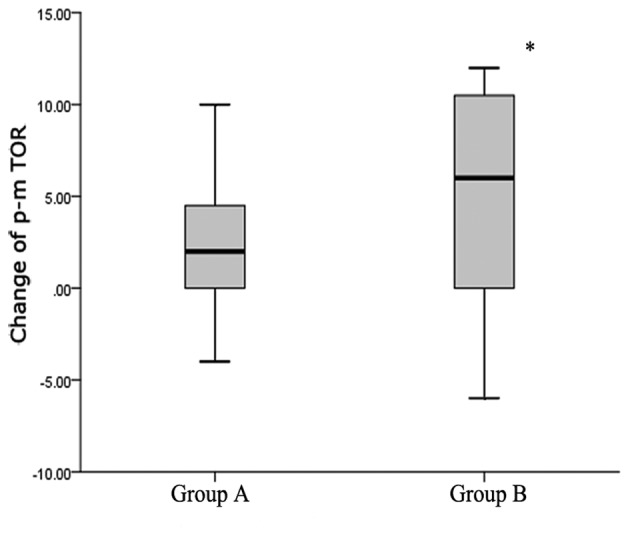
Median values with interquartile ranges of mammalian target of rapamycin changes in groups A and B. Patients whose tumor sizes diminished by <1 cm, showed no change or increased were defined as group A, while patients whose tumor sizes diminished by >1 cm or achieved pathological complete response were defined as group B. The Mann-Whitney U test was used to determine significant differences between groups A and B (P=0.033). The decreased p-mTOR expression was more significant in patients whose tumor size had decreased by >1 cm or who had achieved a complete pathological response.p-mTOR, phosphorylated-mammalian target of rapamycin.

Pre-NAC expression, post-NAC expression and the expression change following NAC of p-mTOR and p-4E-BP1 was evaluated to identify correlations with patient or tumor characteristics, including patient age at diagnosis, tumor grade and stage, estrogen receptor status, and HER2 status of tumors from core biopsy at diagnosis, as well as the tumor stage and presence of axillary metastases from resection pathology. Spearman’s ρ analyses were performed, and not only did the expression of p-mTOR and p-4E-BP1 in pre-NAC samples correlate with HER2 [ρ coefficient, 0.181 (P=0.05) for p-mTOR; ρ coefficient, 0.193 (P=0.04) for p-4E-BP1], which is consistent with the previous study, but the change of mTOR expression was also found to significantly correlate with HER2 (ρ coefficient, 0.275; P=0.006). Next, the changes of p-mTOR in HER2-positive and -negative groups were compared, and the expression of mTOR was found to decrease significantly following treatment with NAC in the HER2-positive group. The changes of p-mTOR expression had median values of 4 in the HER2-positive group and 1 in HER2-negative group. The Mann-Whitney U test was used to assess the differences between the two groups, and a P-value of 0.041 was obtained, suggesting some degree of cross-talk between the PI3K/Akt/mTOR-related pathways and HER2. The expression of p-mTOR and p-4E-BP1 pre- and post-NAC was also evaluated, as well as the expression change of the two factors to identify correlations with tumor size. Therefore, patients were classified into different groups according to tumor size; patients with tumors that had diminished by <1 cm, showed no change, or had increased were defined as group A, while patients whose tumor sizes had diminished by >1 cm, or patients who had achieved PCR were defined as group B. The only significant correlation was found between the expression change of mTOR and diminishing tumor size. The median values of expression change of mTOR were 2 in group A and 6 in group B (Fig. 3). The Mann-Whitney U test showed a significant difference between groups A and B (P=0.033). No significant correlations were found between the two factors and other clinicopathological features.

### High levels of p-mTOR and p-4E-BP1 pre-NAC correlate with poor DFS, and the high expression of p-mTOR post-NAC has a significant association with poor DFS

**Figure 4:**
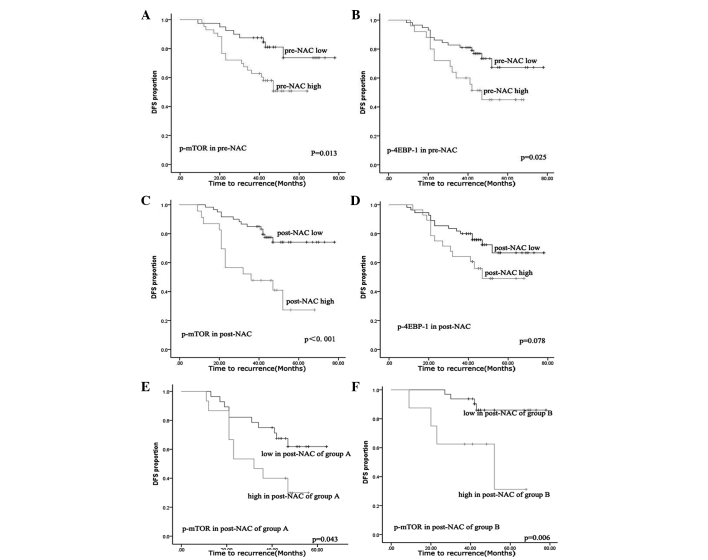
Kaplan-Meier survival analyses of disease-free survival in different patient groups. (A-D) Tumors with high or low expression levels of p-mTOR and p-4E-BP1 pre- and post-NAC. (E and F) Tumors with high or low expression of mTOR post-NAC, in groups A and B. High levels of p-mTOR and p-4E-BP1 expression pre-NAC were found to correlate with a poor DFS, and a high expression of p-mTOR post-NACwas found to be significantly associated with a poor DFS. High expression of p-mTOR post-NAC was found to correlate with a poor DFS, regardless its expression pre-NAC. Group A, high expression of p-mTOR pre-NAC; group B, low expression of p-mTOR pre-NAC; p-mTOR, phosphorylated-mammalian target of rapamycin; p-4E-BP1, phosphorylated-eukaryotic translation initiation factor 4E-binding protein 1; NAC, neoadjuvant; DFS, disease-free survival.

In order to assess the differential survival with respect to pre- and post-NAC expression, as well as the expression change following NAC for the two markers, Kaplan-Meier survival analyses were performed. ROC curve analyses were used to dichotomize the expression scores into high and low expression groups. The cut-off values were obtained from the highest combined sensitivity and specificity at the endpoint of DFS, and were as follows: p-mTOR, 8 and p-4E-BP1, 9 for pre-NAC; and p-mTOR, 7 and p-4E-BP1, 5 for post-NAC. A high expression of p-mTOR and p-4E-BP1 was found to significantly correlate with poor DFS pre-NAC (Fig. 4A and B, log-rank, P=0.013 for p-mTOR and P=0.025 for p-4E-BP1). The expression of p-4E-BP1 post-NAC was not significantly correlated with DFS (Fig. 4D). By contrast, the high expression of p-mTOR in post-NAC samples had a significant association with poor DFS compared with the pre-NAC samples (Fig. 4C, log-rank, P<0.001). It was also examined whether the expression changes of the factors correlate with DFS. Changes in expression of the two factors were dichotomized as up- or downregulated, but no significant correlation was found. In order to identify the predictive value of mTOR post-NAC, patients with high p-mTOR expression pre-NAC were defined as group A, while those with low p-mTOR expression pre-NAC were defined as group B. Next, the p-mTOR expression was compared between groups A and B post-NAC. High expression of p-mTOR post-NAC was found to correlate with poor DFS, regardless of whether the patients were in group A or B (P=0.043 for group A and P=0.006 for group B). Finally, multivariate Cox regression analysis was performed taking into account p-mTOR and p-4E-BP1 expression pre-NAC, p-mTOR expression post-NAC and other clinicopathological features, including tumor grade, receptor status, tumor stage pre-NAC and axillary metastasis. Post-NAC expression of p-mTOR was identified as the only significant factor, with its high expression associated with a hazard ratio of 3.073 (95% confidence interval, 1.4–6.8; P=0.006).

## Discussion

Consistent with previous studies, the expression levels of p-mTOR and p-4E-BP1 in pre-NAC samples were found to significantly correlate with each other in this study. However, following chemotherapy, the expression levels of the two factors were found to decrease and no longer showed a correlation. This may indicate that the PI3K/Akt/mTOR/4E-BP1 pathway can be suppressed by chemotherapy in certain patients without treatment with mTOR inhibitors. This result supports the previous finding that the expression of eIF4E, as the downstream factor of p-4E-BP1, was reduced following NAC (34). This may also suggest that chemotherapy can suppress certain upstream signals or regulators of mTOR, such as Akt, PTEN and TSC1/TSC2, resulting in the inhibition of the mTOR/4E-BP1/eIF4E pathway. HER2-mediated activation of the PI3K/Akt/mTOR pathway has been implicated in the angiogenesis and metastasis of breast cancers (35), and is predictive of tumor progression (2). In an *in vitro* study, HER2-overexpressing cells with an activated Akt/mTOR/4E-BP1 pathway were more dependent on this pathway for growth and, therefore, were more sensitive to mTOR inhibition (2). A previous study has also shown that the expression of p-mTOR and p-4E-BP1 correlate with HER2 expression, which is consistent with the finding in this study. The decrease of p-mTOR was also found to be significant in HER2-positive patients compared with HER2-negative patients, indicating that the PI3K/Akt/mTOR pathway may be suppressed more effectively by chemotherapy in HER2-positive breast cancers. Based on these results, we hypothesize that HER2-positive patients with high p-mTOR expression following NAC may benefit more from the addition of mTOR inhibitors to chemotherapy. However, further study is required to investigate the specific association between the PI3K/Akt/mTOR/4E-BP1 pathway and chemotherapy sensitivity.

It has been demonstrated that patients with favorable responses to NAC exhibit improved DFS (36). Previous clinical trials have also shown that patients who achieve pCR exhibit an improved DFS and OS (16–19). Although a number of different prognostic indicators are being developed, few molecular markers are widely used to predict the NAC responses. In this study, the change of p-mTOR expression was found to correlate with the change of tumor size. Patients with lower levels of p-mTOR expression following NAC are likely to have smaller tumor sizes. Although this finding is not useful to predict the sensitivity of chemotherapy prior to NAC, it can be used as a marker of the effect during the course of NAC and as a reference to decide the chemotherapy regimen following surgery. It was also noted that the PI3K/Akt/mTOR pathway correlates with the resistance to chemotherapy based on the analysis of the correlation between the change of p-mTOR expression and the change of tumor size.

This study not only confirmed previous findings that high levels of p-mTOR and p-4E-BP1 pre-NAC are significantly associated with poor DFS, but also found that the high expression of p-mTOR in post-NAC samples exhibits a significant association with poor DFS. No significant correlation was found between the changes of the two factors, or between the post-NAC expression of 4E-BP1 and DFS. In an effort to investigate the reason why the change of p-mTOR was not found to correlate with DFS, it was demonstrated that the p-mTOR expression significantly correlated with DFS, regardless of whether its expression was high or low pre-NAC. This result indicated that patients whose tumors contain high levels of p-mTOR following NAC may be more resistant to chemotherapy. These patients, particularly HER2-positive patients, may be more appropriate candidates for adding mTOR inhibitors to the chemotherapy. As a downstream factor of p-4E-BP1, eIF4E has been demonstrated to correlate with DFS, regardless of its expression in neoadjuvant (34) or adjuvant chemotherapy (10). However, the p-4E-BP1 expression post-NAC or the change of p-4E-BP1 was not found to correlate with DFS. Although the exact mechanism is not clear, this may be due to the different chemotherapy regimens.

The expression levels of p-mTOR and p-4E-BP1 were significantly decreased following treatment with NAC, particularly in HER2-positive samples. However, little is known with regard to the mechanisms that drive the expression changes of the two factors. The p-mTOR expression post-NAC may be a more reliable predictor to DFS in NAC patients, and may be used as a reference to select patients that are suitable for adding mTOR inhibitors to the chemotherapy. It is known that mutations of PIK3CA, Akt and PTEN, or the loss of PTEN may influence the expression of p-mTOR following NAC. Therefore, these mutations may cause different sensitivities to chemotherapy. Further study is required to clarify the exact mechanisms and to evaluate the predictive value of the PI3K/Akt/mTOR/4E-BP1 pathway in NAC.

## Figures and Tables

**Table I:** Clinical and pathological features of the patients (n=83).

Characteristic	n (%)
Age, years	
≤45	49 (59.0)
>45	34 (41.0)
Pre-NAC stage (based on ultrasound)	
T2	41 (49.4)
T3	24 (28.9)
T4	18 (21.7)
Post-NAC stage (based on resection pathology)	
T0	6 (7.2)
T1	23 (27.7)
T2	33 (39.8)
T3	12 (14.5)
T4	9 (10.8)
Tumor size change^a^	
Increase	8 (9.6)
Decrease	69 (83.1)
pCR	6 (7.2)
NAC regimen	
TP	32 (38.6)
TC	15 (18.1)
CEF	36 (43.4)
Positive axillary metastasis	61 (73.5)
Estrogen receptor-positive	37 (44.6)
Her2-positive	51 (61.4)
Surgery	
Breast conserving	2 (2.4)
Mastectomy	81 (97.6)
Follow-up, months^b^	45 (32–78)
Recurrence	28 (33.7)
Mortality	23 (27.7)

a Change between the initial size at ultrasound and final size at resection pathology;

b Median (range).

NAC, neoadjuvant chemotherapy; pCR, pathological complete response; TP, docetaxel + platinum; TC, platinum + cyclophosphamide; CEF, 5-fluorouracil + epirubicin + cyclophosphamide.
